# Factors predicting the response to growth hormone therapy in Taiwanese patients with Turner syndrome

**DOI:** 10.1186/1687-9856-2013-S1-P48

**Published:** 2013-10-03

**Authors:** Ying-Hua Huang, Fu-Sung Lo, Yang-Hau Van, Hsun-Hui Sui

**Affiliations:** 1 Department of Pediatrics, Kaohsiung Chang Gung Memorial Hospital, Taiwan; 2 Division of Pediatric Endocrinology, Linkou Chang Gung Memorial Hospital, Taiwan; 3 Department of Pediatrics, Taipei Medical Hospital, Taipei, Taiwan

## Background

Turner syndrome (TS) occurs in one 2000-2500 live female births and is associated with short stature, premature ovarian failure, and a range of other phenotypic features. Girls with TS achieve an average adult height 20cm shorter than their mid-parenteral height if they didn’t receive treatment. Growth hormone (GH) treatment was associated with highly significant gains in growth and adult height in girls with TS. The purpose of this study was to evaluate the factors that predicting the response to GH in patients with TS.

## Patients and methods

We performed a retrospective cohort study of clinical and laboratory data of 57 Taiwanese TS children who received growth hormone from Nov 1998 to Nov 2011. Patients’ height and weight were measured before and every three months throughout the GH treatment. Bone age (BA) was evaluated every six to twelve months during and after GH treatment. Conjugated estrogen would be introduced if no spontaneous puberty onset (breast Tanner stage 2) was noted after 13 year of age. 37 of these patients were followed until final height.

## Results

From Nov 1998 to Jan 2011, 57 Turner syndrome patients started GH therapy at the mean age of 11.13 years and the dose of GH was 0.33mg/kg/week. Forty-six patients received estrogen replacement therapy at the mean age of 15.09 years, if indicated. The growth rate before treatment was 4.02cm/year, which increased to 7.04, 5.67, 4.72, 4.29cm/year during the first four years of GH therapy, respectively. The first year growth response was found to be positively correlated with height SDS at start of GH treatment and target height; but negatively correlated with age, height, and BA at start of GH treatment. Final adult height correlated positively with height SDS and Turner height SDS at start of GH treatment and first year growth response. Height gain over projected adult height had negative correlation with Turner height SDS and BA at start of GH treatment; but positive correlation with duration of GH treatment and age at start of puberty (spontaneous or induced).

## Conclusions

There was no difference in baseline characteristic between different karyotype except height velocity and number of spontaneous puberty. As children with GH deficiency, the administration of GH to children with TS results in marked acceleration in linear growth, which is most pronounced during the first year of treatment. A paradoxical finding was noted in our finding: children who were tall (according to TS height SDS) at the start of treatment remained tall till adult height, but their height gain was relatively minor.

